# Digestibility of *Duddingtonia flagrans *chlamydospores in ruminants: *in vitro *and *in vivo *studies

**DOI:** 10.1186/1746-6148-5-46

**Published:** 2009-12-28

**Authors:** Nadia F Ojeda-Robertos, Juan FJ Torres-Acosta, Armín J Ayala-Burgos, Carlos A Sandoval-Castro, Rosa O Valero-Coss, Pedro Mendoza-de-Gives

**Affiliations:** 1 Campus de Ciencias Biológicas y Agropecuarias, FMVZ, Universidad Autónoma de Yucatán, Carretera Mérida-Xmatkuil (km 15,5), Mérida, Yucatán, México; 2 Centro Nacional de Investigación Disciplinaria en Parasitología Veterinaria, INIFAP, Carretera Federal Cuernavaca-Cuautla, (km 11,5), No 8534, Col Progreso, Jiutepec, Morelos, CP 62550, México

## Abstract

**Background:**

The use of *Duddingtonia flagrans *as a tool for the biological control of gastrointestinal nematodes (GIN) is a promising alternative to anthelmintics. The chlamydospores of *D. flagrans *are orally dosed and their thick cell wall gives them the capacity to resist digestion and pass through the gastrointestinal tract (GIT). Chlamydospores reaching the faeces are able to germinate and trap nematode larvae. The efficacy of this control method is based on reducing the numbers of infective larvae leaving the faeces. Techniques have recently been developed for quantifying the numbers of chlamydospores in faeces. As the number of non-digested spores could be relevant in the design and optimization of dosing programmes for the control of GIN infective larvae, the aim of the present study was to estimate the loss of *D. flagrans *chlamydospores during their passage through the ruminant gastrointestinal tract using *in vitro *and *in vivo *techniques.

**Results:**

After *in vitro *rumen digestion, chlamydospore recovery was not different from the quantity originally incubated (undigested spores) (P > 0.05). *In vitro *rumen+abomasum digestion caused nearly 36% loss of the chlamydospores originally incubated (P < 0.05). Germination of chlamydospores classified as viable was 24.3%. Chlamydospores classified as non-viable did not germinate. Rumen digestion resulted in more spore germination (R1 = 35.7% and R2 = 53.3%) compared to no digestion (time 0 h = 8.7%). Subsequent abomasal digestion reduced germination (R1+A = 25%) or stopped it (R2+A = 0%). *In vivo *apparent chlamydospore digestibility in sheep showed a loss of 89.7% of the chlamydospores (P < 0.05).

**Conclusions:**

The loss of chlamydospores was evident under *in vitro *and *in vivo *conditions. Negligible amounts of spores were lost during the *in vitro *rumen digestion. However, *in vitro *rumen+abomasum digestion resulted in a chlamydospore loss of approximately 36%. *In vivo *passage through the sheep GIT resulted in a total loss of 89.7% of the orally administered spores.

## Background

Biological control of gastrointestinal nematodes (GIN) with the nematophagous fungus *Duddingtonia flagrans *is based on the use of oral doses of chlamydospores. Chlamydospores are structures surrounded by a thick cell wall that confers on them resistance to variable environmental conditions [1]. Moreover, the cell wall gives them the capacity to resist digestion and pass through the gastrointestinal tract (GIT), and to be excreted in the faeces, while preserving their capacity to germinate and trap nematode larvae [2-4]. This characteristic gives *D. flagrans *a clear advantage when compared with other nematophagous fungi such as *Arthrobotrys *spp. which are less resistant to digestion [5,6]. Nevertheless, the transit through the GIT may reduce the viability of *D. flagrans *chlamydospores [7]. This was first suspected using *in vitro *digestion studies with an *in vitro *incubation process simulating rumen+abomasum conditions that resulted in a significant loss of the chlamydospore viability [2]. Other workers suggested the existence of significant losses of the total chlamydospore number, without any quantitative evidence of this claim [8,9]. The capacity of chlamydospores to bypass the ruminant digestion process has been based on *in vitro *observations using qualitative assessments of chlamydospore resistance (presence or absence of fungal specific features). However, currently it is possible to count the chlamydospores reaching the faeces [10,11]. Such chlamydospores can be considered as non-digested spores. That information could be relevant in the design and optimization of oral doses for the control of GIN infective larvae. The aim of the present study was to estimate the digestibility of *Duddingtonia flagrans *chlamydospores during their passage through the ruminant GIT using *in vitro *and *in vivo *techniques.

## Methods

### *Duddingtonia flagrans *chlamydospores

A Mexican strain of *D. flagrans *(FTHO-8) was used. Chlamydospores were produced at the Centro Nacional de Investigación Disciplinaria en Parasitología Veterinaria (CENID-PAVET, INIFAP), Jiutepec, Morelos, México. The *D. flagrans *chlamydospores used for the *in vivo *and *in vitro *procedures were obtained from different batches of the same strain. Chlamydospores were quantified using a Neubauer chamber [12]. The different chlamydospore suspension doses were prepared (see below) and stored in plastic centrifuge tubes (15 ml capacity), which were kept under refrigeration (4°C) for 3 to 4 weeks until its use for both *in vitro *and *in vivo *procedures. Viability of spores contained in the respective suspensions was verified prior to their use in the different experimental procedures. For this purpose a qualitative technique was used, which is based on the spore's ability to germinate and to form trapping structures in the presence of *Haemonchus contortus *infective larvae [4].

### Ruminal digestion *in vitro*

*Duddingtonia flagrans *chlamydospores were subjected to an *in vitro *incubation simulating the rumen digestion. Ten doses with increasing quantities of chlamydospores were used (from 6.9 × 10^5 ^to 5.97 × 10^6 ^chlamydospores). The number of chlamydospores in the respective doses was obtained from three counts using a Neubauer chamber [12]. Doses were obtained by diluting a stock of chlamydospores in suspension (6 × 10^6 ^chlamydospores per ml) in buffer. The lowest dilution used 1.5 ml of the stock suspension and 13.5 ml of buffer. The other doses were obtained with 1.5 ml increments of the chlamydospore suspension stock. Buffer was added to achieve a final volume of 15 ml. The highest dose used contained 15 ml of the stock suspension and 0 ml of the buffer. Four replicates were incubated for each dose (n = 40).

Incubations were performed in 40 glass bottles (100 ml capacity) clearly identified with numbers and included 2 g of ground feed (50% maize grain and 50% chopped star grass hay (*Cynodon nlemflensis*)). Rumen incubation medium was prepared with 20 ml of strained rumen liquid, 65 ml buffer [13] and different chlamydospore doses (15 ml). Total incubation media was 100 ml. Two incubation periods were tested: 12 h and 24 h (R1 and R2 respectively) in order to simulate the range of rumen digestion and turnover kinetics likely to be encountered by the chlamydospores [14].

• Chlamydospores at time 0 h: At the beginning of the incubation, the content of each glass bottle was thoroughly mixed to ensure the homogeneous distribution of spores. Then, 5 ml of the suspension were withdrawn to assess the initial chlamydospore count without digestion (time 0 h). This value was used as a reference to evaluate the chlamydospore loss after the incubation processes.

• R1: The remaining 95 ml of suspension contained in the bottles were sealed and incubated in a bacteriological oven at 38°C. After 12 h of incubation, contents were homogenized, the bottles were opened and 5 ml samples were obtained for chlamydospore counting.

• R1+A: Forty ml of the R1 suspensions were transferred to additional flasks for a second digestion process simulating the abomasum digestion (described below). At the end of which, 5 ml samples were obtained for chlamydospore counting.

• R2: The remaining 50 ml from R1 were sealed again and incubated for further 12 h (24 h of total incubation time). After this period, each bottle was thoroughly mixed to obtain 5 ml of the chlamydospore suspension from each bottle to quantify chlamydospores.

• R2+A: The 45 ml left in the flasks from rumen incubation (R2) were transferred to other individual flasks to continue with the abomasal digestion (described below). A 5 ml sample was also obtained at the end of this process.

• All 5 ml samples (Time 0, R1, R2, R1+A and R2+A) were kept under refrigeration (4°C) until the chlamydospores were counted.

### *In vitro *abomasal digestion

The suspensions obtained from the rumen incubations (R1 = 40 ml and R2 = 45 ml) were transferred to Erlenmeyer flasks (50 ml) and HCL (50% v/v) was added until a pH 2.0 was achieved (approximately 3.5 ml). Then, 0.5 ml of pepsin solution (16 mg in 20 ml HCl 0.075 N) was added. Flasks were incubated in a water bath at 39°C. After 4 h incubation, digestion was stopped by adding phosphate buffer (pH 7.5) and bicarbonate to adjust to pH 7.5 [15] (R1+A and R2+A).

### Chlamydospore quantification and quality determination

The forty 5 ml samples from the different digestion procedures were mixed (vortex) to obtain a respective 1 ml aliquots. The counting procedure included time 0 (undigested chlamydospores or control treatment). Each aliquot was diluted in distilled water until a volume of 5 ml was reached. The number of chlamydospores in each aliquot was determined from three independent counts using a Neubauer chamber [12]. During the counting procedure chlamydospores were visually assessed and classified according to their integrity (shape and structure) at 40× magnification. Chlamydospores were classified as a) viable (undisrupted cell wall and with presence of granules), or as b) not-viable (disrupted cell wall or absence of granules).

### Chlamydospore germination capacity

For each *in vitro *incubation time and procedure (0 h, R1, R2, R1+A and R2+A) ten samples of 200 μl were obtained (a total of 50 samples). A technique to obtain single spores from the aliquots [12] in each digestion procedure was used. Undigested chlamydospores (n = 33) were obtained from time 0 h and 24 spores from each of the other incubation times and procedures (R1, R2, R1+A and R2+A). A total of 129 individual chlamydospores were incubated. The spores were classified as viable and non-viable as described above. Each individual chlamydospore was incubated in a water-agar (0.20%) Petri dish. After 8 d, chlamydospore germination was assessed and recorded as positive (with germination and mycelial growth and trapping structures) or negative (no germination) [4].

### *In vivo *chlamydospore apparent digestibility

Data used to determine the *in vivo *chlamydospore apparent digestibility (CAD) were generated in a previous study [10]. The trial was performed in a humane manner consistent with animal welfare considerations valid in Mexico. Animals were not exposed to any stressful condition and no animal was sacrificed or harmed. Chlamydospores were orally administered to nine male growing sheep (mean LW 20 kg) which were kept in individual metabolic crates. Doses ranging from 17.8 × 10^7 ^to 1.95 × 10^7 ^chlamydospores were administered to sheep for seven consecutive days without replication (Table 1). Individual chlamydospore doses were prepared in an oat-molasses mixture (5 g fresh weight) and fed to individual animals. Animals were also fed a complete diet composed of star grass hay, maize grain, soybean meal, sugar cane molasses and mineral supplement (27 g of dry matter (DM) per kg body weight, 16% Crude Protein and 70% DM digestibility). After 3 d, chlamydospore excretion reached a steady state condition, as suggested by the McMaster counts in which spores were counted [10]. Hence, samples from days 3 to 7 (five days) were used to quantify faecal excretion of chlamydospores per gram of faeces. Total quantity of faeces was collected daily from each sheep. A representative sample of 10% of the faeces of each animal was taken to determine its DM. Total faecal excretion per day (dry basis) was estimated for the period evaluated. This value was used to estimate total chlamydospore excretion per day. The chlamydospore apparent digestibility (%) was calculated as:

Where:

CAD = Chlamydospore apparent digestibility

OCD = Oral chlamydospore dose

CRF = Chlamydospores recovered in faeces (number of chlamydospores per g of faeces × total faecal weight)

**Table 1:** *Duddingtonia flagrans *oral chlamydospore dose (OCD) offered per day and chlamydospores recovered in faeces (CRF) per day in sheep used to determine the chlamydospore apparent digestibility (CAD).

Sheep	Dose (OCD)	CRF (mean ± SE)	CAD (%)*
1	1.95 × 10^7^	1.36 × 10^6 ^± 5.76 × 10^5^	93.0
2	2.68 × 10^7^	3.31 × 10^6 ^± 3.42 × 10^5^	87.6
3	3.13 × 10^7^	3.79 × 10^6 ^± 3.91 × 10^5^	87.9
4	4.31 × 10^7^	4.76 × 10^6 ^± 7.14 × 10^5^	89.0
5	6.13 × 10^7^	7.57 × 10^6 ^± 11.5 × 10^5^	87.6
6	6.50 × 10^7^	6.79 × 10^6 ^± 9.86 × 10^5^	89.6
7	9.50 × 10^7^	10.2 × 10^6 ^± 20.2 × 10^5^	89.2
8	12.0 × 10^7^	9.66 × 10^6 ^± 15.0 × 10^5^	91.9
9	17.8 × 10^7^	15.8 × 10^6 ^± 43.3 × 10^5^	91.1
Mean			89.7

*Chlamydospore apparent digestibility (CAD) = (OCD-CFR)/DC*100.

### Statistical analyses

*In vitro *digestibility was determined as the percentage of dosed chlamydospores (time 0 h) not counted after the different digestion procedures (R1, R2, R1+A, R2+A) [16]. As ten graded doses were used for the *in vitro *digestion, digestibility was calculated as explained below.

Data of chlamydospore quantity in each *in vitro *time/procedure (n = 40) was used to calculate the *in vitro *chlamydospore digestibility (IVCD) using the following equation:

Where:

IVCD = *in vitro *chlamydospore digestibility (%)

B_1 _= recovery of undigested chlamydospore (*in vitro*). The B_1 _value was obtained from the following linear regression equation (modified from Schneider and Flat, [17]):

Where:

CR = Chlamydospore recovery at each digestion stage (R1, R2, R1+A and R2+A)

B_0 _= Intercept

B_1 _= Slope (represents the proportional recovery or the undigested chlamydospores)

CDose = chlamydospore doses at 0 h.

The data distribution showed a small standard deviation on the left side of the x axis and a large standard deviation on the right side of the x axis. However, the relative variability was consistent along the x axis. Thus, the regression analysis was performed minimizing the sum of squares of relative distances: Σ [Y_data _- Y_curve_/Y_data_]^2 ^[18] using GraphPad Prism 4.0. The regression lines (slopes) of the different digestion procedures were compared using that same programme.

Additionally, the quantity of chlamydospores found at time 0 h (40 counts) was compared to that in R1, R2, R1+A and R2+A (40 counts at each digestion procedure) using separate Mann-Whitney tests (Minitab Inc. Release 12).

For the *in vivo *chlamydospore apparent digestibility data, means (and standard errors of the mean) of the chlamydospores recovered in faeces are reported.

## Results

### *In vitro *ruminal and abomasal digestibility

**Figure 1:**
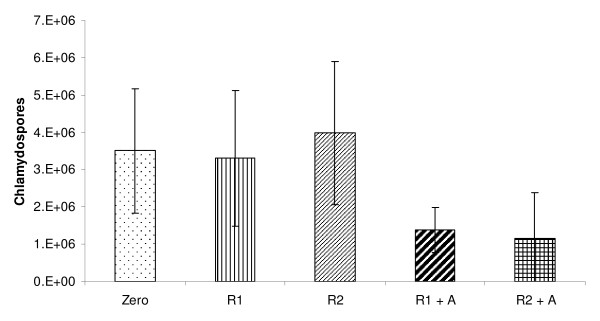
**Mean (+- SD) chlamydospore quantity before incubation (time 0 h), after *in vitro *rumen digestion (12 h (R1) and 24 h (R2)) and after 4 h of *in vitro *abomasal digestion (R1+A and R2+A)**.

The chlamydospores (mean ± SD) recovered in each digestion process are presented in Figure 1. After the 12 and 24 hour rumen digestion, the CR was similar to the number originally incubated (P > 0.05). The spore recovery (B_1 _*100) after ruminal digestion was 96% (r^2 ^= 0.94) and 88% (r^2 ^= 0.92) for R1 and R2, respectively.

The number of chlamydospores recovered after both rumen + abomasum digestions processes (R1+A and R2+A) was significantly lower than those in time 0 h (P < 0.01). After the ruminal+abomasal incubation, the chlamydospore recovery was 69% (r^2 ^= 0.77) and 58% (r^2 ^= 0.12) for the R1+A and R2+A, respectively.

The regression lines of spore counts after ruminal incubations (R1 *vs*. R2) were not significantly different. Also, the regression lines of R1+A and R2+A were not different. Hence, data were pooled to generate single regression lines for each digestion phase.

The resulting pooled equations were:

and

From the regression lines of spore counts it can be stated that a similar quantity of chlamydospores was recovered after ruminal incubations compared to that at time 0 h. On the other hand, the *in vitro *apparent digestibility for the rumen + abomasum equation was estimated to be 35.63% (Rumen + Abomasum CR).

### Chlamydospores germination capacity

From the 129 individual chlamydospores cultivated in water agar Petri dishes, 20 were classified as non-viable and 109 as viable. A total of 36 chlamydospore cultures were discarded from further studies due to contamination (31 viable and 5 non-viable chlamydospores). Germination and growth was only found on chlamydospores classified as viable. However, the germination observed was 24.3% (19/78). On the other hand, all the chlamydospores classified as non-viable did not germinate irrespective of time and digestion procedure. Chlamydospores obtained at time 0 h were all classified as viable but only 8.7% germinated (2/23). Interestingly, the proportion of germinated chlamydospores increased after the rumen digestion procedure, where R1 showed a 35.7% germination (5/14) and R2 53.3% (8/15). Germination was reduced after both abomasal digestion procedures (R1+A with 25% and R2+A 0% germination).

The largest quantity of non-viable chlamydospores was obtained from R2+A (11/20).

### *In vivo *digestibility of chlamydospores

The *in vivo *apparent digestibility data of experimental sheep is presented in table 1. Digestibility values were within a range of 87.6 to 93% with an average of 89.7 (± SE 0.65). Digestibility was observed along the range of doses employed and no trend was detected suggesting higher or lower digestibility as a result of higher or lower spore oral doses.

## Discussion

The use of *D. flagrans *as a biological control agent against GIN infective larvae was based on experimental evidence which identified nematophagous fungal species capable of resisting/withstanding *in vitro *digestion, thereby simulating the passage through the ruminant GIT [2]. Such a crucial experiment used a qualitative assessment of the germination capacity of *D. flagrans *and *Arthrobotrys *spp. It also enabled scientists to identify *D. flagrans *chlamydospores as a structure that resisted digestion in the GIT. However, no quantitative assessment was attempted.

In spite of the considerable scientific progress associated with the use of *D. flagrans *chlamydospores [19], the percentage of chlamydospores that were lost *in vivo *during transit through the GIT remained unknown. Larsen et al. [2,7] suggested that chlamydospores had a reduced viability due to their transit through the GIT. However, it is important to point out that both viability and recovery of chlamydospores in the faeces need to be considered for effective usage of *D. flagrans*. Faedo et al. [8] and Grønvold et al. [9] suggested a significant loss (not measured) of orally administered chlamydospores as a result of the passage through the GIT (digestion). Their suggestions were not confirmed in quantitative terms. However, the reduction in the number of spores due to digestion in the GIT seems to be an important factor in explaining the performance of *D. flagrans *when used *in vivo *[10,11].

### *In vitro *digestibility

In the present study, *in vitro *rumen digestion of *D. flagrans *chlamydospores (FTHO-8 strain) was negligible. However, the abomasal acid digestion (pepsin + HCl) did cause the loss (digestion) and damage of the fungal structures as evaluated by a reduction of its germination capacity and the presence of more non-viable chlamydospores. The regression equations obtained with data from the present trial indicate a 9% loss (P > 0.05) after rumen digestion and a further 36% loss (P < 0.05) after abomasal digestion. The results agreed with the qualitative observation of Larsen et al. [2] who stated that pepsin digestion caused more stress than rumen liquor to *D. flagrans *structures.

### Chlamydospores germination capacity

Chlamydospores classified as non-viable did not germinate. This observation confirms the validity of the criteria used to judge damaged chlamydospores. From those chlamydospores classified as viable, 24.3% germinated. There is no published information available to compare with the present results on the germination of viable *D. flagrans *spores. It is possible that the large amount of chlamydospores produced by the fungi already takes into consideration a low germination capacity as a part of its reproductive strategy.

However, the need remains to confirm whether the 75.6% of the so-called "viable" chlamydospores that did not germinate were actually non-viable or incorrectly classified by the criteria used. If the latter is true, additional criteria need to be developed to assess viability.

The lower germination capacity in the chlamydospores obtained at time 0 h and the higher germination obtained after the rumen digestion procedures (R1 and R2) suggested the possibility of a beneficial effect of rumen digestion on chlamydospores. Rumen digestion might cause the scarification of spores resulting in improved germination. However, the nil germination rate obtained from viable chlamydospores enduring 24 hours of rumen digestion plus abomasal digestion (R2+A) could indicate that chlamydospores might be damaged in a manner not detectable by the procedure established in the present trial. In general, the R2+A digestion procedure would mimic an unusually long rumen retention time which is not common in small ruminants [20]. Furthermore, the small particle size of chlamydospores would suggest that a shorter rumen retention time is more likely [10].

### *In vivo *apparent digestibility

The present report is an attempt to assess, in a quantitative manner, the *in vivo *apparent digestibility of *D. flagrans *chlamydospores through the gastrointestinal tract of sheep.

The chlamydospores recovered in the faeces after an oral dosage undergo the complete digestive processes in the GIT (rumen, abomasum, small and large intestine). As a consequence, on average, 89.7% of the chlamydospores orally administered were not recovered in the faeces. The present result matches the values suggested by Grønvold et al. [9] where they speculated that more than 90% of dosed chlamydospores were lost in the GIT. Similarly, Grønvold et al. [5] and Faedo et al. [8] suggested a high loss of chlamydospores but no quantitative assessment was made. Under the conditions of diet and animals of the present trial, the amount of chlamydospores reaching the faeces was around 10% irrespective of the dose used.

### Fate of chlamydospores in the GIT

Summarizing the present results, and assuming an additive nature of the digestion processes, the destruction of *D. flagrans *chlamydospores along the GIT can be described as a non significant loss in the rumen (up to 9%) and up to 36% digestion after both rumen and abomasal digestion. The *in vivo *studies showed that only 10% of an oral dose (irrespective of the dose used) would be recovered in the faeces under the dietary conditions of the present experiment. The latter highlights the need to complete the information of this trial by challenging chlamydospores to *in vitro *rumen, abomasum and also small intestine digestion processes in a consecutive manner as performed in the present trial. This could help to confirm whether the *in vitro *digestibility can predict the *in vivo *apparent digestibility of *D. flagrans *spores. Such trial should include the same batch of *D. flagrans *chlamydospores of the same strain for the *in vitro *and *in vivo *procedures to achieve a more accurate comparison.

The present results may be useful for the design of oral doses aimed to obtain a suitable ratio of chlamydospores/nematode eggs in the faeces and, therefore, better effectiveness on the use of *D. flagrans *for GIN control.

## Conclusions

A negligible proportion of chlamydospores are lost during *in vitro *rumen digestion (nearly 9%). However, *in vitro *abomasal digestion resulted in 36% loss. *In vivo *passage along sheep GIT resulted in a mean loss of 89.7% of the orally administered spores. These results confirm a significant loss of chlamydospores which should be taken into account to design oral doses and to explain the variable efficacy of *D. flagrans *against infective larvae.

## Authors' contributions

NFOR, CASC, JFJTA: Design of the study; NFOR: Logistics of the trial, incubations and sample collections; NFOR, ROVC: chlamydospore counting (oral doses and post incubation counts), spore germination procedures; NFOR, ROVC, CASC, JFJTA, AJAB, PMG: data analyses, statistics and writing the scientific paper.
